# Accessory Ossicles in the Region of the Foot and Ankle: An Epidemiologic Survey in a Jordanian Population

**DOI:** 10.3390/medicina57111178

**Published:** 2021-10-29

**Authors:** Heba Kalbouneh, Omar Alajoulin, Jamil Shawaqfeh, Ayman Mustafa, Shehab Jaber, Shaima’ Zaben, Ja’far Zapen, Mohammad Alsalem

**Affiliations:** 1 Department of Anatomy, School of Medicine, The University of Jordan, Amman 11942, Jordan; m_alsalem@ju.edu.jo; 2 Orthopedic and Trauma Department, Jordanian Royal Medical Services, Amman 11855, Jordan; Omaralajoulin@gmail.com; 3 Radiology Department, Jordanian Royal Medical Services, Amman 11855, Jordan; jshawaqfeh@yahoo.com; 4 Department of Basic Medical Sciences, College of Medicine, QU Health, Qatar University, Doha 2713, Qatar; amustafa@qu.edu.qa; 5 Biomedical and Pharmaceutical Research Unit, QU Health, Qatar University, Doha 2713, Qatar; 6 School of Medicine, The University of Jordan, Amman 11942, Jordan; shehabbjabeer@gmail.com (S.J.); sym0182452@ju.edu.jo (S.Z.); Jaf0193487@ju.edu.jo (J.Z.)

**Keywords:** accessory ossicle, incidence, foot radiology, Jordanians, ankle, anatomy

## Abstract

*Background and Objectives*: The incidence of accessory bones in the region of foot and ankle is quite variable between studies and are often confused with avulsion fractures in trauma patients with musculoskeletal injuries. The aim of this study was to assess the incidence of accessory ossicles of the foot and ankle according to gender, side and coexistence, and to determine how frequently accessory ossicles were misdiagnosed as avulsion fractures. *Materials and Methods*: Oblique and/or lateral foot radiographs of 1000 adult patients referred from emergency departments to foot and ankle clinic were retrospectively reviewed for the presence of accessory ossicles. The Kappa statistic was used in order to assess the validity of radiographic interpretation for the presence of these bones. *Results*: Accessory ossicles were detected in 40.2% of the radiographs. The incidence rates for the accessory ossicles in order of frequency were: Os trigonum (15.4%), accessory navicular (13.7%), os peroneum (11.5%), os vesalianum (1.1%), os supranaviculare (0.7%), os subfibulare (0.6%), os talotibiale (0.4%), os calcaneus secundarius (0.3%), os supratalare (0.3%), os infranaviculare (0.3%), os intermetatarseum (0.2%), and os subtibiale (0.1%). Coexistence of two or three ossicles in the same foot was observed in 4.4% of the cases, mostly coexistence with os peroneum (2.9%), followed by accessory navicular (1.6%). 2.7% of accessory ossicles were initially misdiagnosed as avulsion fractures at emergency departments. Interrater agreement over identification of different accessory ossicles was found to be reasonably reliable, with a Kappa greater than 0.80 for all assessed bones. *Conclusions*: In clinical practice, a thorough knowledge of normal anatomical variants is essential to facilitate appropriate diagnosis and treatment and can help to prevent diagnostic errors.

## 1. Introduction

The accessory ossicles are small, well-corticated supernumerary osseous structures that are frequently encountered in the region of the foot and ankle. They could be ovoid or nodular, unilateral or bilateral, bipartite or multipartite, and may be present near a bone or an articulation [1]. Accessory bones originate from ossification centers that have failed to fuse with the main bone [2,3]. They are considered developmental anomalies and can occur either bilaterally or unilaterally.

Accessory ossicles of the foot are detected incidentally by radiological examinations. They are generally of little clinical significance and are rarely associated with painful syndromes. However, they are increasingly being reported in the literature due to the pain directly related to them, especially following overuse and trauma, the restricted range of motion, or the misinterpretation of them as fractures [1,4]. Diagnostic imaging is necessary to deliver pertinent information that has to be considered in the workup of the patients.

Due to excessive stresses and strains encountered in the emergency departments, clinical practice is prone to diagnostic error [5]. Some of emergency physicians’ referrals to orthopedic surgeons are deemed unnecessary and represent a significant financial burden to the patients and the health care system [6]. Accessory bones are often confused with avulsion fractures in trauma patients with musculoskeletal injuries. Given the potential for misinterpretation, recognizing the normal variants that present as uncommon accessory bones of the foot and ankle is essential for emergency physicians to facilitate correct diagnosis and treatment and to avoid unnecessary referral of the patients [7,8]. There is an extensive variability in the reported prevalence of the accessory ossicles in the region of foot and ankle in the international literature. Moreover, there are no published studies on the validation of radiographic assessment for the presence of these ossicles, particularly the rarest ones. The aim of this study was to determine the incidence of accessory ossicles of the foot and ankle in a large group of Jordanian individuals according to gender, side, and coexistence, and to determine how frequently accessory ossicles were misdiagnosed as avulsion fractures. Additionally, we aimed to assess the validity of the interpretation of their radiographic findings as a measure for the detection of these bones.

## 2. Materials and Methods

A retrospective review of anteroposterior, oblique and/or lateral foot radiographs of 1000 adult patients referred from emergency departments to foot and ankle clinic following trauma between September 2013 and September 2020 was performed. This study was approved by the Institutional Review Board at Jordanian Royal Medical Services and the need for informed consent was waived due to the retrospective nature of this study.

The inclusion criteria were patients aged 18 years and older and the availability of high-quality foot radiographs that demonstrated the ankle region and all tarsal and metatarsal bones. Radiographs were of a single foot from each patient, either right or left. Data on patient age, gender, and foot laterality (right vs. left) were recorded from hospital records. All radiographs were reviewed by two independent investigators and the data were recorded in order to assess the validity of the radiographic interpretation in identifying the accessory ossicles. Disagreements were resolved via discussion to reach a consensus or a third investigator was consulted to avoid any discrepancy.

Statistical Analysis: GraphPad Prism version 6.04 for Windows (GraphPad Software, La Jolla, CA, USA) was used. The incidence of different accessory ossicles was compared between genders (male vs. female) and sides (right vs. left) using Fisher’s exact test. The significance threshold was set at 0.05. Interobserver reliability was analyzed using the kappa statistic to assess the consistency between two investigators in identifying the accessory ossicles. The 95% confidence interval and standard error of the kappa were calculated.

## 3. Results

Radiographic views of 674 right and 326 left feet from 1000 adult patients (500 males and 500 females) were included in this study. The mean age (±SD) was 36.5 years ±16.9 years.

Incidence of accessory ossicles in the foot and ankle region was shown in Table 1. Accessory ossicles were detected in 40.2% of the radiographs (402/1000). Except for os trigonum, no significant differences in the proportion of accessory ossicles were observed between sexes or sides (*p* < 0.05). The incidence rate of os trigonum was statistically higher among females (*p* < 0.05). In males, accessory ossicles were found in 41.6% (208/500) of the cases. In females, accessory ossicles were found in 38.8% (194/500) of cases. Accessory ossicles were seen in 42.8% (289/674) of the right feet and 38.9% (127/326) of the left feet (Table 1).

**Figure 1:**
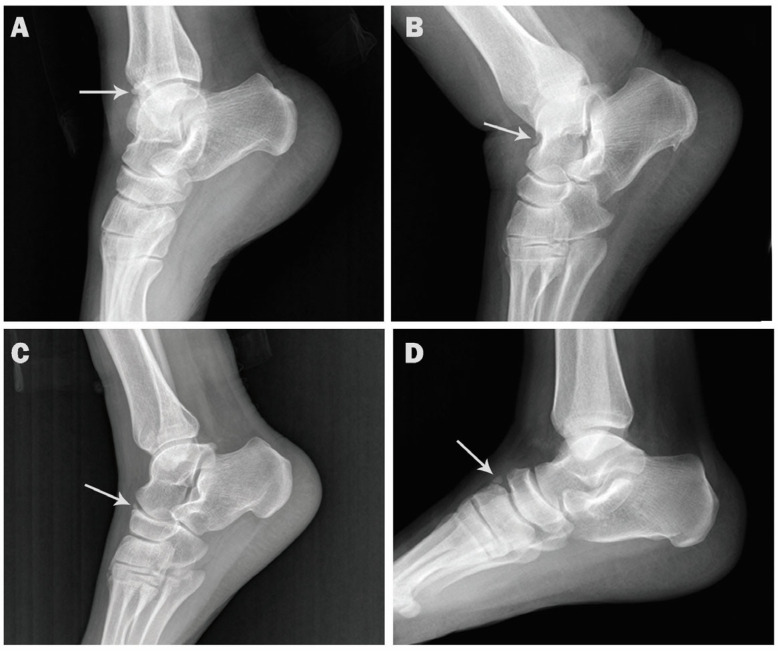
Lateral radiographs showing accessory ossicles (arrows). (**A**). Os talotibiale in a 38-year-old female. (**B**). Os supratalare in 47-year-old male. (**C**). Os supranaviculare in 51-year-old male. (**D**). Os infranaviculare in 25-year-old male.

**Figure 2:**
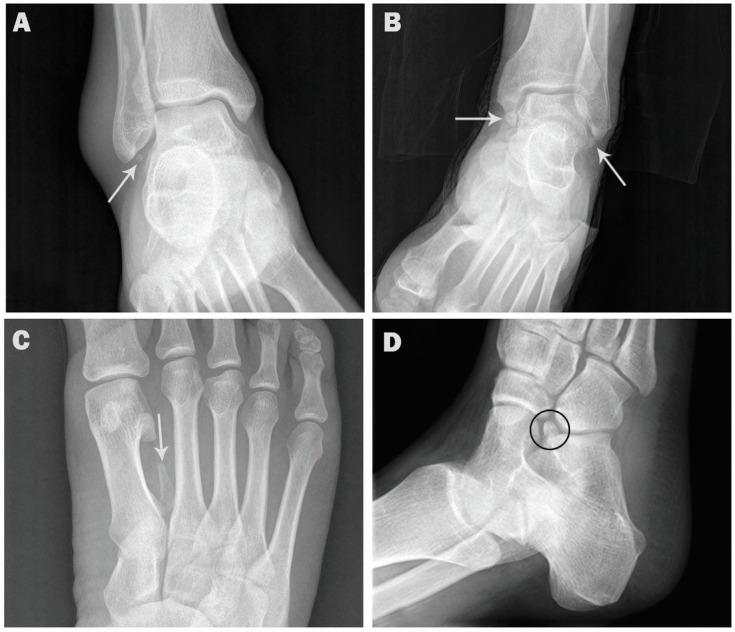
AP and oblique radiographs showing accessory ossicles. (**A**). AP ankle radiograph showing os subfibulare in a 53-year-old male (arrow). (**B**). AP ankle radiograph showing os subfibulare and os subtibiale in a 40-year-old male (arrows). (**C**). AP foot radiograph showing os intermetatarseum in 27-year-old female (arrow). (**D**). Oblique radiograph showing os calcaneus secundarius in 29-year-old female (circle).

The incidence rates for the accessory ossicles in order of frequency were: Os trigonum (15.4%), accessory navicular (13.7%), os peroneum (11.5%), os vesalianum (1.1%), os supranaviculare (0.7%), os subfibulare (0.6%), os talotibiale (0.4%), os calcaneus secundarius (0.3%), os supratalare (0.3%), os infranaviculare (0.3%), os intermetatarseum (0.2%), and os subtibiale (0.1%) (Figure 1 and Figure 2).

**Figure 3:**
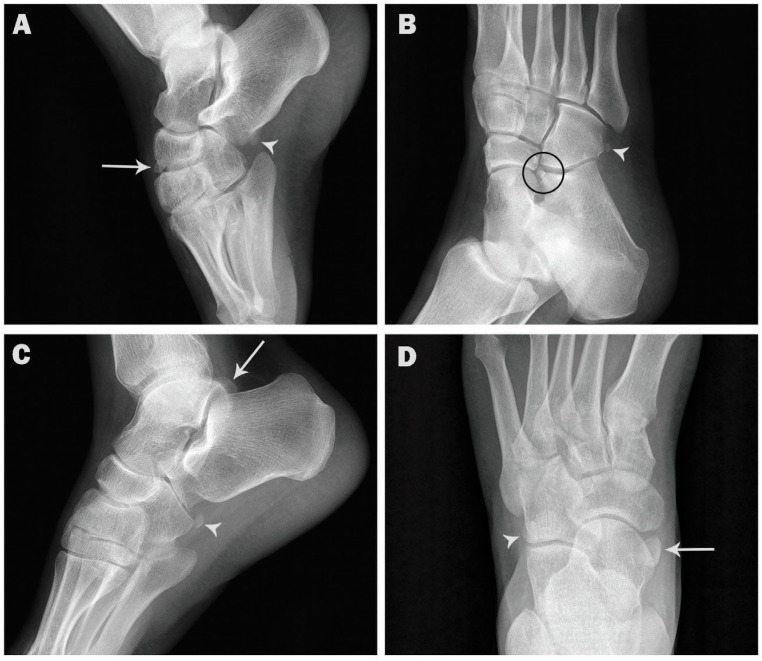
Coexistence of os peroneum and other ossicles in the same foot. (**A**). Lateral radiograph showing os peroneum (arrowhead) and os infranaviculare (arrow) in a 31-year-old female. (**B**). Oblique radiograph showing os peroneum (arrowhead) and os calcaneus secundarius (circle) in a 52-year-old female. (**C**). Lateral radiograph showing os peroneum (arrowhead) and os trigonum (arrow) in a 48-year-old male. (**D**). AP radiograph showing os peroneum (arrowhead) and accessory navicular (arrow) in a 55-year-old male.

**Figure 4:**
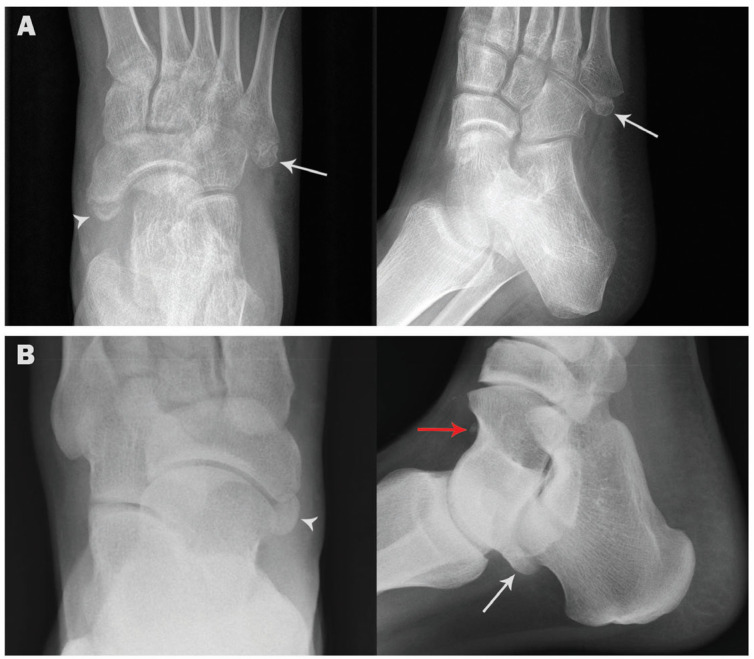
Coexistence of accessory navicular and other ossicles in the same foot. (**A**). AP and oblique radiographs showing accessory navicular (arrowhead) and os vesalianum (arrow) in a 56-year-old female. (**B**). AP and lateral radiographs showing accessory navicular (arrowhead), os trigonum (white arrow) and os supratalare (red arrow) in a 45-year-old male.

**Figure 5:**
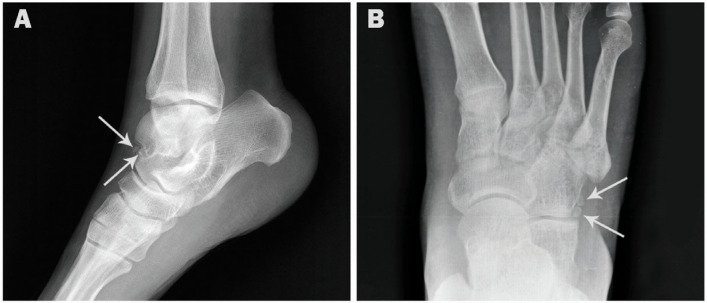
Radiographs showing bipartite ossicles (arrows). (**A**). Lateral radiograph showing bipartite supratalare in a 44-year-old female. (**B**). AP radiograph showing bipartite os peroneum in 50-year-old male.

Coexistence of two or three ossicles in the same foot was observed in 44 cases (4.4%), mostly coexistence with os peroneum (29 cases, 2.9%) (Figure 3), followed by accessory navicular (16 cases, 1.6%) (Figure 4). Coexistence of os subfibulare and os subtibiale was noted in only one case (0.1%) (Figure 2C). Multipartite ossicles were observed in 0.9% of the radiographs (9/1000); bipartite os peroneum was observed in eight cases of and a bipartite supratalare in one case (Figure 5).

Kappa statistics on interobserver agreement are shown in Table 2. Interobserver agreement over identification of different accessory ossicles was almost perfect (kappa > 0.9) except for os supratalare, os subtibiale, os intermetatarseum, os calcaneus secundarius, and os infranaviculare, the agreement was substantial (kappa between 0.61 and 0.80).

Os calcanei accessorium, os sustentaculum, os tali accessorium, os cuboideum secundarium, os paracuneiforme, os intercuneiforme, os cuneometatarsale I tibiale, os cuneo-I metatarsale-I plantare, os cuneo-I metatarsale-II dorsale, os aponeurosis plantaris, and os subcalcis were not found in this survey. We observed an incidence of 2.7% of accessory ossicles misdiagnosed as avulsion fractures (11/402). After 73 patients with incomplete medical records had been excluded, none of the patients had clinical symptoms related to these ossicles. 

## 4. Discussion

The reported incidence of the accessory ossicles in the region of the foot and ankle ranges from 18.3% to 36.3% in general populations [4,8,9]. A higher incidence (40.3%) of these ossicles was reported in our dataset. One of the reasons could be that in this study the patients were referred by emergency departments, and misinterpretation of accessory ossicles as fragments of avulsion fractures is a common occurrence. However, a high incidence of accessory ossicles was also reported in a healthy asymptomatic population. In a recent Korean population study, accessory ossicles were found in 49.2% of the healthy, asymptomatic Korean adults [10]. Additionally, in a CT-based study, accessory bones were detected in 47.5% of the scans. The high incidence rate of the accessory bones in the later study could be explained by the use of CT which is more sensitive than plain radiography in detecting these osseous structures [11].

The accessory navicular bone, os peroneum and os trigonum are the most common reported ossicles in different studies [8,11]. The accessory navicular is located on the medial side of the foot, proximal to the navicular and continuous with the tibialis posterior tendon. The frequency of occurrence of this ossicle in incidental X-rays varies from 2 to 20.2%% among general populations [7,9,12]. Os peroneum is embedded within the peroneus longus tendon. It is related to the plantar or lateral surface of the cuboid. Os peroneum may be in an ossified, cartilaginous, or fibrocartilaginous state [13]. The ossified form is seen in up to 32% of feet [8,14]. Os trigonum is located at the posterior aspect of the talus adjacent to the lateral tubercle. The reported incidence of os trigonum ranges between 2 and 50% [15,16].

In this study, the most common accessory ossicle in the foot was os trigonum (14.4%), followed by accessory navicular (12.7%) and os peroneum (10.5%). However, in radiographic studies from Turkey and Japan, accessory navicular was the most common accessory bone [9,17]. On the other hand, many studies reported the accessory navicular as the second most frequently occurring accessory bone after the os peroneum and ahead of os trigonum [1,8,18]. The incidence rates of os trigonum, accessory navicular and os peroneum in our subjects were higher than the rates of 2.3%, 11.7% and 4.7% reported by Coskun et al. [9], but lower than the 23.5%, 28.2% and 31.8% reported by Cilli et al. [8]. Additionally, in our previous studies, the frequency of occurrence for os trigonum and accessory navicular was considerably higher in comparison with the current study (26.1% and 22.9%, respectively) [19,20]. One of the reasons could be that CT scans were used for os trigonum detection, which have higher sensitivity for detecting accessory ossicles compared to conventional radiographs. For accessory navicular, symptomatic patients with medial foot pain were included; therefore, the incidence of accessory navicular was considerably higher than the incidence obtained in this study.

On the other hand, the accessory ossicles: os intermetatarseum, os calcaneus secundarius, os supratalare, os vesalianum, os subfibulare, os supranaviculare, os infranaviculare, os talotibiale, os subfibulare, and os subtibiale were rarely reported in the literature [1,9,11]. In our population, the low incidence of these accessory bones was almost consistent with previous reports. The os intermetatarseum is observed between the medial cuneiform and the base of the first and second metatarsals. This ossicle originates from the distal corner of the medial cuneiform, tapers distally, and projects between the first and second metatarsals [21]. It may be difficult to differentiate this ossicle from fractures of the base of the second metatarsal or calcified dorsalis pedis artery [22]. It has an estimated prevalence of 1.2–10%. Os intermetatarseum had a lower frequency of occurrence in our study, as it was observed in only 0.2% of the radiographs.

The os calcaneus secundarius is located in an interval between the anteromedial aspect of the calcaneus, the proximal aspect of the cuboid and navicular, and the talar head [23,24]. It may be difficult to differentiate from a fracture of the anterior process of the calcaneus. It is observed in up to 5% of the population [25]. In this study, os calcaneus secundarius was observed in only 0.3% of the radiographs.

The os supratalare, also known as talus secundarius, is typically located superior to the head or neck of talus between the ankle and talonavicular joints [22]. Os supratalare may be fused with the talus or remain as a free ossicle. Os supratalare can be confused with os talotibiale, os supranaviculare, an avulsion fracture or osteoarthritic degeneration of the talonavicular joint [21]. It has an estimated prevalence of 0.2% to 2.4% [9,17,21,22]. The prevalence of os supratalare in our study was 0.3%.

Os vesalianum is located near the base of the fifth metatarsal bone and is found within the peroneus brevis tendon at its insertion, with an estimated incidence of 0.1 and 5.9% [17,22]. Os vesalianum can be confused with acute avulsion fracture of the fifth metatarsal bone, Jones fracture, Iselin’s disease, and os peroneum [22]. Os vesalianum was found in 1.1% of our series.

The os subfibulare is located beneath the tip lateral malleolus. It can reach up to 10 mm in diameter and can be confused with an avulsion fracture of the lateral malleolus [21]. The incidence of os subfibulare represented on radiography ranges between 0.2% and 6.6% in the general population [17,26]. In our series, os subfibulare was found in 0.6%.

The os supranaviculare is located at the superior margin of the navicular, in the area of the talonavicular joint, close to the midpoint. It may be fused with the talus or with the navicular and may be confused with an avulsion fracture of navicular bone, osteoarthritic degeneration of the talonavicular joint, or os supratalare. However, os supratalare is located superior to the head or neck of talus. Os supranaviculare presents in 1% and 3.5% of the population [8,17,27]. However, a lower prevalence for os supranaviculare (0.7%) was reported in this study.

The os infranaviculare is known also as cuneonavicular ossicle, it is located at the inferior margin of the navicular between the navicular, medial cuneiform, and intermediate cuneiform bones, usually overriding the medial cuneiform [28]. Incidence of this ossicle is poorly documented in the international literature. Os infranaviculare was observed in 0.3% of our cases.

The os subtibiale is located beneath the medial malleolus on its posterior aspect and possibly results from an unfused ossification center at the distal tip of the medial malleolus [29]. It should be differentiated from a fracture of medial malleolus. According to previous reports, the incidence of os subtibiale ranges between 0.2% and 2.1% [17,29,30,31]. Os subtibiale was observed in 0.1% of our cases.

Os talotibiale is situated in front of the tibiotalar joint and may predispose to anterior ankle impingement syndrome. It is estimated to present in 0.5% of general population [22]. Os talotibiale was observed in 0.4% of our cases. The large differences in the incidence of accessory ossicles in the foot and ankle as cited in different reported surveys can be attributed to several factors; in particular, the frequency of occurrence may differ based on the method of evaluation (cadaveric, plain radiography, or CT), and/or the patient characteristics (incidental findings vs. symptomatic feet). Additionally, it may be attributed to the interethnic differences, intergenetic factors, and mechanical stresses [7,32].

## 5. Conclusions

In conclusion, the incidence of the accessory ossicles in the current study did not differ greatly from the previous literature. Although accessory ossicles are rarely associated with painful syndromes, it is important to understand their typical locations and appearances. This study may be helpful to assist the clinicians in recognizing the normal variants that present as rare accessory bones of the foot and ankle, and to avoid misinterpreting them as fragments of avulsion fractures. A thorough knowledge of normal anatomical variants is essential to facilitate appropriate diagnosis and treatment and to avoid diagnostic error and unnecessary workup of the patients.

## Figures and Tables

**Table 1:** Incidence of the accessory ossicles in the region of the foot and ankle.

Accessory Ossicle	Incidence (%) N = 1000	Male (%) N = 500	Female (%) N = 500	Right (%) N = 674	Left (%) N = 326
All	402 (40.2)	208 (41.6)	194 (38.8)	289 (42.8)	127 (38.9)
*p* value		0.1214		0.2454	
Coexistence of two or three ossicles	44 (4.4)	24 (4.8)	20 (4.0)	28 (4.1)	16 (4.9)
*p* value		0.6442 ^ns^		0.6226 ^ns^	
Os trigonum	154 (15.4)	65 (13.0)	89 (17.8)	105 (15.6)	49 (15.0)
*p* value		0.0436 *		0.8522 ^ns^	
Accessory navicular	137 (13.7)	77 (15.4)	60 (12.0)	99 (14.7)	38 (11.7)
*p* value		0.1410 ^ns^		0.2033 ^ns^	
Os peroneum	115 (11.5)	64 (12.8)	51 (10.2)	81 (12.0)	34 (10.4)
*p* value		0.2341 ^ns^		0.5260 ^ns^	
Os intermetatarseum	2 (0.2)	1 (0.2)	1 (0.2)	2 (0.3)	0 (0)
*p* value		1.000 ^ns^		1.000 ^ns^	
Os vesalianum	11 (1.1)	7 (1.4)	4 (0.8)	9 (1.3)	2 (0.6)
*p* value		0.5466 ^ns^		0.5187 ^ns^	
Os calcaneus secundarius	3 (0.3)	2 (0.4)	1 (0.2)	2 (0.3)	1 (0.3)
*p* value		1.000 ^ns^		1.000 ^ns^	
Os supratalare	3 (0.3)	2 (0.4)	1 (0.2)	1 (0.1)	2 (0.6)
*p* value		1.000 ^ns^		0.2493 ^ns^	
Os subfibulare	6 (0.6)	2 (0.4)	4 (0.8)	3 (0.4)	3 (0.9)
*p* value		0.6866 ^ns^		0.3979 ^ns^	
Os supranaviculare	7 (0.7)	6 (1.2)	1 (0.2)	4 (0.6)	3 (0.9)
*p* value		0.1237 ^ns^		0.6886 ^ns^	
Os infranaviculare	3 (0.3)	2 (0.4)	1 (0.2)	2 (0.3)	1 (0.3)
*p* value		1.000 ^ns^		1.000 ^ns^	
Os subtibiale	1 (0.1)	1 (0.2)	0 (0)	0 (0)	1 (0.3)
*p* value		1.000 ^ns^		0.3260 ^ns^	
Os talotibiale	4 (0.4)	3 (0.6)	1 (0.2)	2 (0.3)	2 (0.6)
*p* value		0.6242 ^ns^		0.6001 ^ns^	

* *p* ≤ 0.05, Fisher Exact Test. ^ns^ not significant

**Table 2:** Interobserver reliability (Kappa) for the detection of accessory ossicles.

		95% Confidence Interval		
Accessory Ossicle	Kappa	Lower	Upper	SE
All	0.956	0.937	0.975	0.010
Os trigonum	0.975	0.955	0.995	0.010
Accessory navicular	0.986	0.971	1.000	0.008
Os peroneum	0.989	0.974	1.000	0.008
Os intermetatarseum	0.800	0.415	1.000	0.196
Os vesalianum	0.841	0.663	1.000	0.091
Os calcaneus secundarius	0.800	0.415	1.000	0.196
Os supratalare	0.666	0.229	1.000	0.223
Os subfibulare	0.909	0.730	1.000	0.091
Os supranaviculare	0.832	0.604	1.000	0.117
Os infranaviculare	0.800	0.415	1.000	0.196
Os subtibiale	0.666	0.050	1.000	0.314
Os talotibiale	0.857	0.579	1.000	0.142

## Data Availability

The datasets used and/or analyzed during the present study are available from the corresponding author on reasonable request.
